# Non-contiguous finished genome sequence and description of *Alistipes timonensis* sp. nov.

**DOI:** 10.4056/sigs.2685971

**Published:** 2012-07-20

**Authors:** Jean-Christophe Lagier, Fabrice Armougom, Ajay Kumar Mishra, Thi-Tien Nguyen, Didier Raoult, Pierre-Edouard Fournier

**Affiliations:** 1 Unité de Recherche sur les Maladies Infectieuses et Tropicales Emergentes, UMR, Aix-Marseille Université, Marseille, France

**Keywords:** *Alistipes timonensis*, genome

## Abstract

*Alistipes timonensis* strain JC136^T^ sp. nov. is the type strain of *A. timonensis* sp. nov., a new species within the genus *Alistipes*. This strain, whose genome is described here, was isolated from the fecal flora of a healthy patient. *A. timonensis* is an obligate anaerobic rod. Here we describe the features of this organism, together with the complete genome sequence and annotation. The 3,497,779 bp long genome (one chromosome but no plasmid) contains 2,742 protein-coding and 50 RNA genes, including three rRNA genes.

## Introduction

*Alistipes timonensis* strain JC136^T^ (= CSUR P148 = DSM 25383) is the type strain of *A. timonensis* sp. nov. This bacterium is a Gram-negative, anaerobic, indole-positive bacillus and was isolated from the stool of a healthy Senegalese patient as part of a “culturomics” study aiming at cultivating individually all species within human feces.

With more than 3,000 genome sequences available, bacterial genomics has revolutionized several aspects of microbiology. To date, taxonomy has remained unaffected by this progress, despite the debate around the definition of bacterial species. Despite its elevated cost, poor reproducibility and inter-laboratory comparability, DNA-DNA hybridization remains the “gold standard” criterion [1]. Even the application of internationally validated cutoff values in 16S rRNA sequence similarity that enabled the taxonomic classification or reclassification of hundreds of taxa, is debated [2]. High throughput genome sequencing and mass spectrometric analyses of bacteria provide access to a wealth of genetic and proteomic information [3]. We propose to use a polyphasic approach [4] to describe new bacterial taxa that includes their genome sequence, MALDI-TOF spectrum and main phenotypic characteristics (habitat, Gram-stain reaction, culture and metabolic characteristics, and when applicable, pathogenicity).

Here we present a summary classification and a set of features for *A. timonensis* sp. nov. strain JC136^T^ together with the description of the complete genomic sequencing and annotation. These characteristics support the circumscription of the species *A. timonensis*.

The genus *Alistipes* (Rautio *et al*. 2003) was created in 2003 [5]. To date, this genus, composed of bile-resistant, strictly anaerobic and Gram-negative bacilli, contains five species including *A. finegoldii* (Rautio *et al*. 2003) [5], *A.indistinctus* (Nagai *et al.* 2010) [6], *A. onderdonkii* (Song *et al.* 2006) [7], *A. putredinis* (Weinberg *et al.* 1937) Rautio *et al.* 2003 [5], and *A. shahii* (Song *et al.* 2006) [7]. Pigment production, initially considered as characteristic of *Alistipes* species, was recently demonstrated to be inconstant [8]. Members of the genus *Alistipes* are members of the normal human intestinal microbiota, but have also been reported in urine and the mouth [7], and have occasionally been isolated from abdominal, appendiceal and rectal abscesses, blood cultures from colon cancer patients [9], and feces from children with irritable bowel syndrome [10]. *A. putredinis* was also demonstrated to be associated to cruciferous vegetable intake [11]. In addition, *A. finegoldii* has been suspected to play the role of growth promoter in chickens [12].

## Classification and features

**Figure 1:**
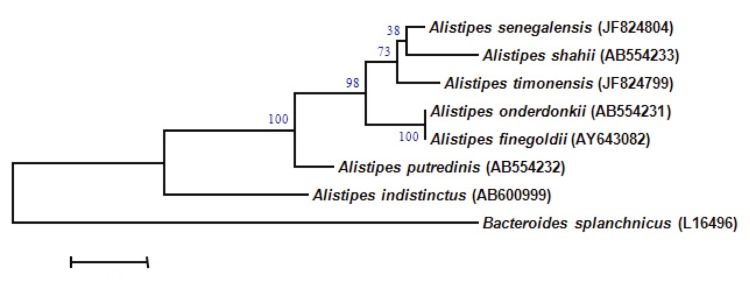
Phylogenetic tree highlighting the position of *Alistipes timonensis* strain JC136^T^ relative to other type strains within the *Alistipes* genus. GenBank accession numbers are indicated in parentheses. Sequences were aligned using CLUSTALW, and phylogenetic inferences obtained using the maximum-likelihood method within the MEGA software. Numbers at the nodes are percentages of bootstrap values obtained by repeating the analysis 500 times to generate a majority consensus tree. *Porphyromonas asaccharolytica* was used as an outgroup. The scale bar represents a 2% nucleotide sequence divergence.

A stool sample was collected from a healthy 16-year-old male Senegalese volunteer patient living in Dielmo (a rural village in the Guinean-Sudanian zone in Senegal), who was included in a research protocol. The patient gave an informed and signed consent, and the agreement of the National Ethics Committee of Senegal and the local ethics committee of the IFR48 (Marseille, France) were obtained under agreement 09-022. The fecal specimen was preserved at -80°C after collection and sent to Marseille. Strain JC136 (Table 1) was isolated in June 2011 by anaerobic cultivation on 5% sheep blood-enriched Columbia agar (BioMerieux, Marcy l’Etoile, France). This strain exhibited 96.98% and 98.13% nucleotide sequence similarities with *A. shahii* (Song *et al.* 2006) and *A. senegalensis* (Mishra *et al.* 2012), respectively, the phylogenetically closest validated *Alistipes* species (Figure 1) [7]. This value was lower than the 98.7% 16S rRNA gene sequence threshold recommended by Stackebrandt and Ebers to delineate a new species without carrying out DNA-DNA hybridization [2]. It should be noted that both *A. senegalensis* strain JC50T and strain JC136 were cultivated from the same individual.

**Table 1:** Classification and general features of *Alistipes timonensis* strain JC136^T^

**MIGS ID**	**Property**	**Term**	**Evidence code^a^**
		Domain *Bacteria*	TAS [13]
		Phylum *Bacteroidetes*	TAS [14,15]
		Class *Bacteroidia*	TAS [14,16]
	Current classification	Order *Bacteroidales*	TAS [14,17]
		Family *Rikenellaceae*	TAS [14,18]
		Genus *Alistipes*	TAS [5,19]
		Species *Alistipes timonensis*	IDA
		Type strain JC136^T^	IDA
	Gram stain	Negative	IDA
	Cell shape	Bacilli	IDA
	Motility	Nonmotile	IDA
	Sporulation	Nonsporulating	IDA
	Temperature range	Mesophile	IDA
	Optimum temperature	37°C	IDA
MIGS-6.3	Salinity	Growth in BHI medium + 1% NaCl	IDA
MIGS-22	Oxygen requirement	Anaerobic	IDA
	Carbon source	Unknown	NAS
	Energy source	Unknown	NAS
MIGS-6	Habitat	Human gut	IDA
MIGS-15	Biotic relationship	Free living	IDA
MIGS-14	Pathogenicity Biosafety level Isolation	Unknown 2 Human feces	NAS
MIGS-4	Geographic location	Senegal	IDA
MIGS-5	Sample collection time	September 2010	IDA
MIGS-4.1	Latitude	13.7167	IDA
MIGS-4.1	Longitude	16.4167	IDA
MIGS-4.3	Depth	Surface	IDA
MIGS-4.4	Altitude	51 m above sea level	IDA

Evidence codes - IDA: Inferred from Direct Assay; TAS: Traceable Author Statement (i.e., a direct report exists in the literature); NAS: Non-traceable Author Statement (i.e., not directly observed for the living, isolated sample, but based on a generally accepted property for the species, or anecdotal evidence). These evidence codes are from the Gene Ontology project [20]. If the evidence is IDA, then the property was directly observed for a live isolate by one of the authors or an expert mentioned in the acknowledgements.

**Figure 2:**
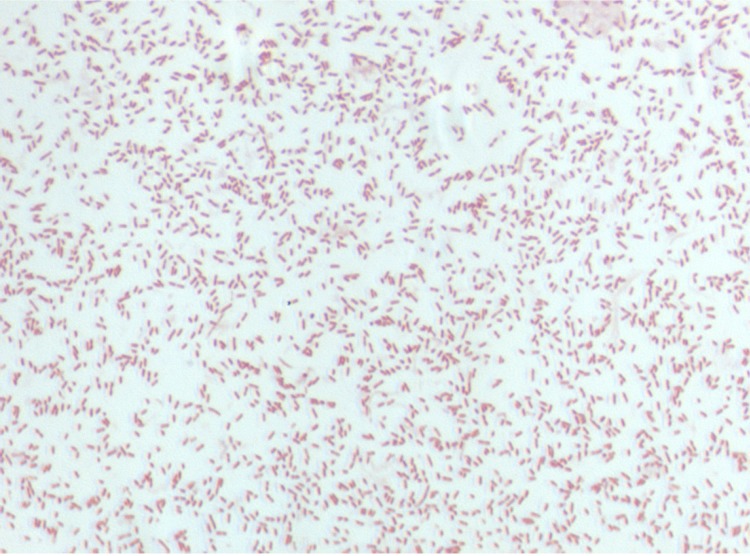
Gram staining of *A. timonensis* strain JC136^T^

**Figure 3:**
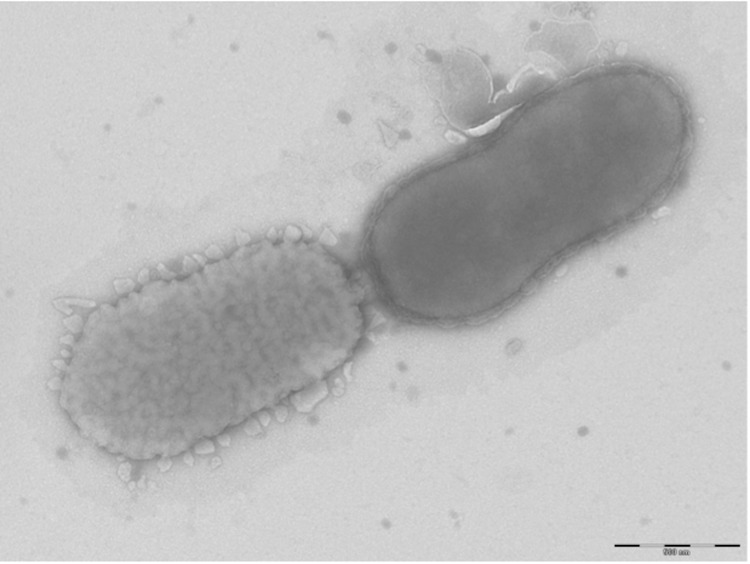
Transmission electron microscopy of *A. timonensis* strain JC136^T^, using a Morgani 268D (Philips) at an operating voltage of 60kV. The scale bar represents 900 nm.

Different growth temperatures (25, 30, 37, 45°C) were tested; no growth occurred at 25°C and 45°C, growth occurred at 30°C, and optimal growth was observed at 37°C. Colonies were 0.2 mm to 0.3 mm in diameter on blood-enriched Columbia agar and Brain Heart Infusion (BHI) agar. Growth of the strain was tested under anaerobic and microaerophilic conditions using GENbag anaer and GENbag microaer systems, respectively (BioMerieux), and in the presence of air, with or without of 5% CO_2_, and in aerobic conditions. Optimal growth was achieved anaerobically. No growth was observed in aerobic, microaerophilic and 5% CO_2_ atmospheres. Gram staining showed Gram negative rods (Figure 2). A motility test was negative. Cells grown on agar have a mean diameter of 0.62 µm (Figure 3) and produce brown pigment.

Strain 136^T^ exhibited catalase activity but no oxidase activity, and was resistant to 20% bile. Using API Rapid ID 32A, a positive reaction was obtained for α-galactosidase, β-galactosidase, β-glucuronidase, glutamic acid decarboxylase, leucyl glycine arylamidase and alanine arylamidase. Weak reactions were obtained for indole production and N-acetyl-β-glucosaminidase. No mannose and raffinose fermentation were observed. *A. timonensis* is susceptible to penicillin G, amoxicillin + clavulanic acid, imipeneme, clindamycin, metronidazole and resistant to vancomycin. By comparison with *A. senegalensis*, strain 136^T^ differed in mannose fermentation and proline arylamidase, arginine arylamidase and glycine arylamidase. By comparison with *A. shahii*, strain 136^T^ differed in catalase activity and mannose and raffinose fermentation [7].

**Figure 4:**
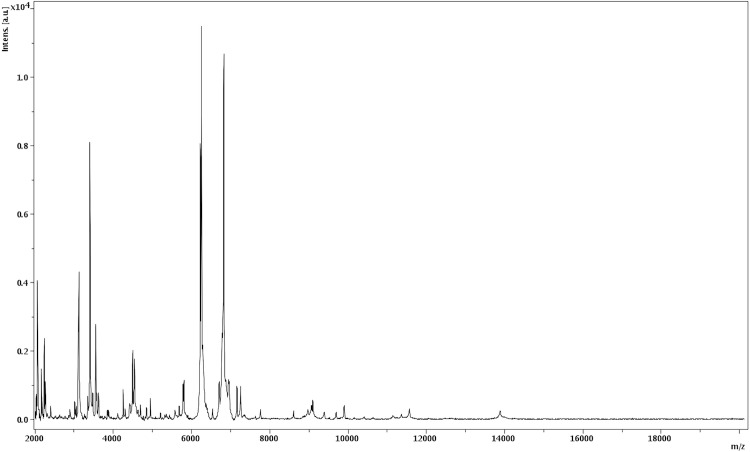
Reference mass spectrum from *A. timonensis* strain JC136^T^. Spectra from 4 individual colonies were compared and a reference spectrum was generated.

Matrix-assisted laser-desorption/ionization time-of-flight (MALDI-TOF) MS protein analysis was carried out as previously described [21]. Briefly, a pipette tip was used to pick one isolated bacterial colony from a culture agar plate, and to spread it as a thin film on a MTP 384 MALDI-TOF target plate (Bruker Daltonics, Leipzig, Germany). Four distinct deposits were done for strain JC136 from four isolated colonies. Each smear was overlaid with 2µL of matrix solution (saturated solution of alpha-cyano-4-hydroxycinnamic acid) in 50% acetonitrile, 2.5% tri-fluoracetic-acid, and allowed to dry for five minutes. Measurements were performed with a Microflex spectrometer (Bruker). Spectra were recorded in the positive linear mode for the mass range of 2,000 to 20,000 Da (parameter settings: ion source 1 (IS1), 20 kV; IS2, 18.5 kV; lens, 7 kV). A spectrum was obtained after 675 shots at a variable laser power. The time of acquisition was between 30 seconds and 1 minute per spot. The four JC136 spectra were imported into the MALDI BioTyper software (version 2.0, Bruker) and analyzed by standard pattern matching (with default parameter settings) against the main spectra of 2,843 bacteria including the spectra from *A. finegoldii*, *A. onderdonkii* and *A. shahii*, used as reference data, in the BioTyper database. The method of identification included the m/z from 3,000 to 15,000 Da. For every spectrum, 100 peaks at most were taken into account and compared with spectra in the database. A score enabled the identification, or not, from the tested species: a score > 2 with a validated species enabled the identification at the species level, a score > 1.7 but < 2 enabled the identification at the genus level; and a score < 1.7 did not enable any identification. For strain 136, the obtained score was 1.2, thus suggesting that our isolate was not a member of a known species. We incremented our database with the spectrum from strain JC136 (Figure 4). The spectrum was made available online in our free-access URMS database [22].

## Genome sequencing information

### Genome project history

The organism was selected for sequencing on the basis of its phylogenetic position and 16S rRNA similarity to other members of the genus *Alistipes*, and is part of a “culturomics” study of the human digestive flora aiming at isolating all bacterial species within human feces. It was the third genome of an *Alistipes* species and the first genome of *Alistipes timonensis* sp. nov. A summary of the project information is shown in Table 2. The EMBL accession number is CAEG00000000 and consists of 23 contigs. Table 2 shows the project information and its association with MIGS version 2.0 compliance [5].

**Table 2:** Project information

**MIGS ID**	**Property**	**Term**
MIGS-31	Finishing quality	High-quality draft
MIGS-28	Libraries used	One paired end 3-kb library and one Shotgun library
MIGS-29	Sequencing platforms	454 GS FLX Titanium
MIGS-31.2	Fold coverage	20×
MIGS-30	Assemblers	Newbler version 2.5.3
MIGS-32	Gene calling method	Prodigal
	EMBL ID	CAEG00000000
	EMBL Date of Release	February 28, 2012
	Project relevance	Study of the human gut microbiome

### Growth conditions and DNA isolation

*A. timonensis* sp. nov. strain JC136^T^, CSUR P148, DSM 25383, was grown anaerobically on 5% sheep blood-enriched Columbia agar at 37°C. Eight petri dishes were spread and resuspended in 4×100µl of G2 buffer (EZ1 DNA Tissue kit, Qiagen, Hilden, Germany). A first mechanical lysis was performed by glass powder on the Fastprep-24 device (MP Biomedicals, Santa Ana, CA, USA) using 2×20 seconds cycles. DNA was then treated with 2.5 µg/µL lysozyme for 30 minutes at 37°C and extracted using the BioRobot EZ 1 Advanced XL (Qiagen). The DNA concentration was measured at 40 ng/µL using the Genios fluorometer (Tecan, Lyon, France).

### Genome sequencing and assembly

Both a shotgun and 3-kb paired-end sequencing were performed. The shotgun library was constructed with 500 ng of DNA with the GS Rapid library Prep kit (Roche). For the paired-end sequencing, 5 µg of DNA was mechanically fragmented on a Hydroshear device (Digilab, Holliston, MA, USA) with an enrichment size at 3-4kb. The DNA fragmentation was visualized using the 2100 BioAnalyzer (Agilent, Massy, France) on a DNA labchip 7500 with an optimal size of 3.393 kb. The library was constructed according to the 454 GS FLX Titanium paired-end protocol. Circularization and nebulization were performed and generated a pattern with an optimal size of 423 bp. After PCR amplification through 15 cycles followed by double size selection, the single stranded paired-end library was then quantified using the Genios fluorometer (Tecan) at 205 pg/µL. The library concentration equivalence was calculated as 8,87E+08 molecules/µL. The library was stored at -20°C until further use.

The shotgun and paired-end libraries were clonally-amplified with 3 cpb and 1cpb, respectively, in 2×8 emPCR reactions with the GS Titanium SV emPCR Kit (Lib-L) v2 (Roche). The yields of the emPCR were 9.3% and 8.9%, respectively. For each sequencing method, approximately 340,000 beads were loaded on the GS Titanium PicoTiterPlate PTP Kit 70×75 and sequenced with the GS FLX Titanium Sequencing Kit XLR70 (Roche). The run was performed overnight and then analyzed on the cluster through the gsRunBrowser and Newbler assembler (Roche). A total of 201,692 passed filter wells were obtained and generated 70.71 Mb with a length average of 325 bp. The passed filter sequences were assembled using Newbler with 90% identity and 40 bp as overlap. The final assembly identified 9 scaffolds and 23 contigs (>1,500bp).

### Genome annotation

Open Reading Frames (ORFs) were predicted using Prodigal [23] with default parameters but the predicted ORFs were excluded if they were spanning a sequencing GAP region. The predicted bacterial protein sequences were searched against the GenBank database and the Clusters of Orthologous Groups (COG) databases using BLASTP. The tRNAScanSE tool [24] was used to find tRNA genes, whereas ribosomal RNAs were found by using RNAmmer [25] and BLASTn against GenBank. ORFans were identified if their BLASTP *E*-value was lower than 1e-03 for alignment length greater than 80 amino acids. If alignment lengths were smaller than 80 amino acids, we used an *E*-value of 1e-05. Such parameter thresholds have already been used in previous works to define ORFans. To estimate the mean level of nucleotide sequence similarity at the genome level between *Alistipes* species, we compared the ORFs only using BLASTN and the following parameters: a query coverage of > 70% and a minimum nucleotide length of 100 bp.

## Genome properties

**Figure 5:**
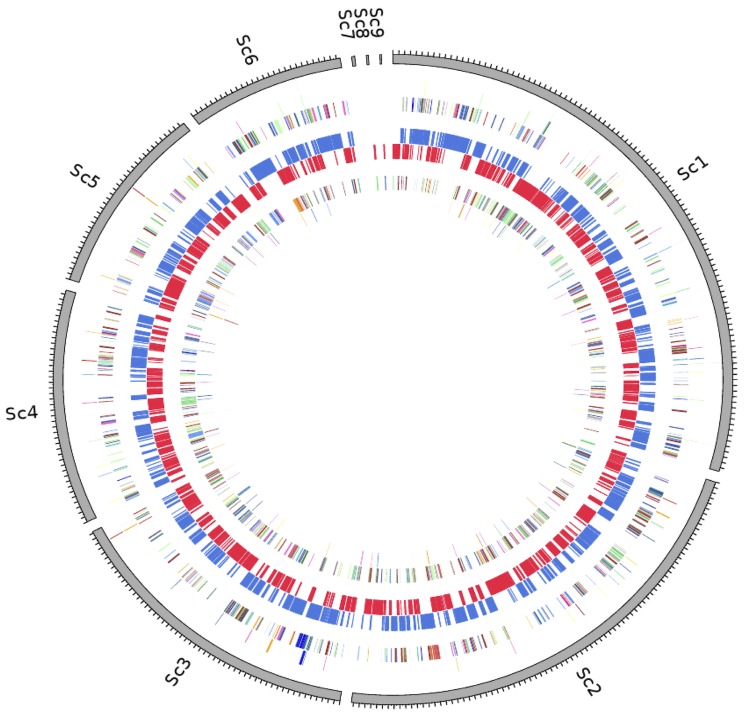
Graphical circular map of the chromosome. From outside to the center: Genes on forward strand (colored by COG categories), genes on reverse strand (colored by COG categories), RNA genes (tRNAs green, rRNAs red), GC content, and GC skew.

The genome is 3,497,779 bp long (one chromosome, no plasmid) with a 58.82% GC content (Table 3, Figure 5). Of the 2,742 predicted genes, 2,692 were protein-coding genes, and 50 were RNAs. A total of 1,885 genes (70.02%) were assigned a putative function. Seventy-eight genes were identified as ORFans (2.9%). The remaining genes were annotated as hypothetical proteins. The distribution of genes into COGs functional categories is presented in Table 4. The properties and the statistics of the genome are summarized in Tables 3 and 4.

**Table 3:** Nucleotide content and gene count levels of the genome

**Attribute**	**Value**	**% of total^a^**
Genome size (bp)	3,497,779	
DNA coding region (bp)	3,232,590	92.42
DNA G+C content (bp)	2,057,393	58.82
Total genes	2,742	100
RNA genes	50	2.0
Protein-coding genes	2,692	98.2
Genes with function prediction	1,885	70,0
Genes assigned to COGs	1,723	64.0
Genes with peptide signals	630	23.4
Genes with transmembrane helices	564	20.9

a) The total is based on either the size of the genome in base pairs or the total number of protein coding genes in the annotated genome.

**Table 4:** Number of genes associated with the 25 general COG functional categories

**Code**	**Value**	**%age**	**Description**
J	136	5.05	Translation, ribosomal structure and biogenesis
A	0	0	RNA processing and modification
K	124	4.60	Transcription
L	92	3.42	Replication, recombination and repair
B	0	0	Chromatin structure and dynamics
D	18	0.67	Cell cycle control, mitosis and meiosis
Y	0	0	Nuclear structure
V	31	1.15	Defense mechanisms
T	86	3.19	Signal transduction mechanisms
M	191	7.10	Cell wall/membrane biogenesis
N	6	0.22	Cell motility
Z	0	0	Cytoskeleton
W	0	0	Extracellular structures
U	32	1.19	Intracellular trafficking and secretion
O	62	2.30	Posttranslational modification, protein turnover, chaperones
C	112	4.16	Energy production and conversion
G	188	6.98	Carbohydrate transport and metabolism
E	141	5.24	Amino acid transport and metabolism
F	54	2.01	Nucleotide transport and metabolism
H	82	3.04	Coenzyme transport and metabolism
I	47	1.74	Lipid transport and metabolism
P	132	4.90	Inorganic ion transport and metabolism
Q	14	0.52	Secondary metabolites biosynthesis, transport and catabolism
R	234	8.69	General function prediction only
S	96	3.57	Function unknown
-	969	35.99	Not in COGs

The total is based on the total number of protein coding genes in the annotated genome.

## Comparison with other *Alistipes* genomes

To date, the complete genomes from *A. senegalensis* strain JC50^T^ (GenBank accession number CAHI00000000), *A. shahii* strain WAL 8301 (GenBank accession number FP929032) and the unfinished genome from *Alistipes sp.* strain HGB5 (AENZ00000000) are available. *A. timonensis* has a smaller genome than *A. senegalensis* and *A. shahii* but a bigger genome than *Alistipes sp.* strain HGB5 (3,497,779 bp *vs* 4,017,609, 3,763,317 bp and 3,464,615, respectively), a higher number of genes than *A. shahii* but smaller than *A. senegalensis* and *Alistipes sp.* strain HGB5 (2,742 *vs* 2,563, 3,163 and 2,955 genes, respectively), a higher ratio of genes assigned to COGs (64.00% *vs* 58.56%, 58.9% and 62.53%, respectively), and a higher G+C content (58.82% *vs* 57.33%, 58.4% and 57%, respectively). In addition, *A. timonensis* shared mean nucleotide sequence similarities at the genome level of 92.18% (range 72.16 to 100%), 88.72% (range 77.86 to 100%) and 85.9% (range 77.4 to 100%), with *A. senegalensis* strain JC50^T^, *A. shahii* strain WAL 8301 and *Alistipes sp.* strain HGB5, respectively.

## Conclusion

On the basis of phenotypic, phylogenetic and genomic analyses, we formally propose the creation of *Alistipes timonensis* sp. nov. that contains the strain JC136^T^. This bacterium has been cultivated from an healthy Senegalese individual, from whom was also cultivated *A. senegalensis* strain JC50^T^, thus suggesting that the fecal flora from humans may contain several undescribed bacterial species that may be isolatable through diversification of culture conditions.

### Description of *Alistipes timonensis* sp. nov.

*Alistipes timonensis* (tim.on.en’sis. L. gen. masc. n. *timonensis*, of Timone, the name of the hospital where strain JC136^T^ was isolated).

Colonies are 0.2 to 0.3 mm in diameter and produce brown pigment on blood-enriched Columbia agar and Brain Heart Infusion (BHI) agar. Cells are rod-shaped with a mean diameter of 0.62 µm. Optimal growth is achieved anaerobically. No growth is observed in aerobic or microaerophilic conditions. Growth occurs between 30-37°C, with optimal growth observed at 37°C, in BHI medium + 5% NaCl. Cells stain Gram negative and are non-motile. Catalase, α-galactosidase, β-galactosidase, β-glucuronidase, glutamic acid decarboxylase, leucyl glycine arylamidase, N-acetyl-β-glucosaminidase and alanine arylamidase activities are present. Indole production is also present. Oxidase activity is absent. Cells are susceptible to penicillin G, amoxicillin + clavulanic acid, imipeneme and clindamycin and metronidazole. The G+C content of the genome is 58.82%. The 16S rRNA and genome sequence are deposited in GenBank under accession numbers JF824799 and CAEG00000000, respectively.

*A. timonensis* is an obligate anaerobic Gram-negative bacterium. Grows on axenic medium at 37°C in an anaerobic atmosphere. Not motile.

The type strain JC136^T^ (= CSUR P148 = DSM 25383) was isolated from the fecal flora of a healthy patient in Senegal.
